# Personality type profiles of medical students and their differences by gender, age, and academic level in Korea: a cross-sectional study

**DOI:** 10.3352/jeehp.2026.23.7

**Published:** 2026-04-28

**Authors:** Yera Hur, Sanghee Yeo

**Affiliations:** 1 Institute of Medical Education, College of Medicine, Hallym University, Chuncheon, Korea; 2 Department of Medical Humanities and Medical Education, School of Medicine, Kyungpook National University, Daegu, Korea; 3 Kyungpook National University Hospital, Daegu, Korea; Hallym University, Korea

**Keywords:** Personality, Medical students, Psychological tests, Educational measurement, Mentoring, Republic of Korea

## Abstract

**Purpose:**

Understanding the psychological characteristics of contemporary medical students is essential for effective educational design and learner support. This study aimed to identify medical students’ personality types using a geometric personality assessment tool (GEOPIA), determine whether differences exist by gender, age, or academic level, and explore the practical utility of such profiling for supporting educational practices in medical school settings.

**Methods:**

The 40-item Korean Geometric Psychological Assessment (GEOPIA) was administered to 1,173 students across 5 Korean medical schools. GEOPIA classifies individuals into 4 primary types—Round (sociable, relationship-oriented), Triangle (task-oriented, challenging), Box (prudent, stability-seeking), and Curve (creative, sensitive). Frequency analyses and χ^2^ tests were conducted. Of the 1,016 respondents (response rate, 86.61%), 981 were included in the final analysis.

**Results:**

The most common primary type was Round (40.3%), followed by Box (31.7%), Triangle (15.2%), and Curve (12.8%). Across the 12 combined profiles, Round–Box (21.9%) was the most prevalent, followed by Box–Round (19.0%) and Round–Triangle (9.7%). No significant differences were observed by gender (χ^2^=6.360, P=0.095, Cramer’s V=0.082), age (χ^2^=11.454, P=0.490, Cramer’s V=0.065), or academic level (χ^2^=18.044, P=0.260, Cramer’s V=0.078).

**Conclusion:**

GEOPIA may provide a practical tool for identifying learner characteristics and supporting educational decision-making in medical school settings. In instructional design, personality-type data can inform group formation, activity planning, and assignment structure. In student support, the tool offers instructors and advisors a quick way to understand learners’ characteristics, which may help guide individualized counseling and promote effective learning experiences.

## Graphical abstract

[Fig f1-jeehp-23-07]

## Introduction

### Background

Identifying learner characteristics is one of the fundamental considerations in educational design aimed at enhancing educational effectiveness. Understanding the psychological characteristics of medical students is especially important for learner-centered educational design and student support. Because medical training involves substantial academic and emotional demands, personality differences may influence how students engage with learning environments and manage stress. Previous studies have shown that such characteristics are associated with learning behaviors, communication, academic performance, and professional development in medical education [1-5].

#### Personality assessment in medical education

A growing body of literature highlights the relevance of personality profiling for understanding medical students’ adaptation to training environments, approaches to learning, and capacity to cope with stress. Systematic reviews indicate broad interest in assessing personality traits, behavioral styles, and emotional intelligence among health care trainees and professionals as part of efforts to support learner development and enhance educational quality [6]. Research among medical students shows that personality profiles are related to well-being, resilience, and academic attitudes, highlighting the value of accessible assessment tools [7, 8]. As institutions expand learner-centered curricula, tailored advising, and student support services, practical and culturally appropriate methods for profiling students’ psychological tendencies have become increasingly necessary.

#### GEOPIA as a brief personality profiling tool

To address the need for brief and practical personality profiling tools in educational contexts, the Korean Geometric Psychological Assessment (GEOPIA) was developed as a structured instrument based on 4 geometric archetypes. Unlike earlier projective psychogeometric approaches, GEOPIA uses a standardized item-based format that enables rapid assessment and straightforward interpretation. To establish the validity and reliability of GEOPIA, its relationship with the Myers-Briggs Type Indicator (MBTI), a scientifically grounded personality assessment tool, has been examined. Findings indicate that higher scores on the circle and S-shaped (curvilinear) types are associated with the Feeling (F) and Perceiving (P) dimensions, whereas higher scores on the triangle and square types correspond to the Thinking (T) and Judging (J) dimensions. In addition, higher scores on the circle and triangle types are related to Extraversion (E), while higher scores on the square and S-shaped types are associated with Introversion (I). These results demonstrate meaningful associations between GEOPIA and the widely used MBTI personality framework, thereby supporting the reliability of the GEOPIA instrument [9]. Furthermore, GEOPIA has been shown to have high applicability in areas such as career exploration and counseling [10].

Unlike conventional assessment tools that require respondents to complete numerous questionnaire items, GEOPIA enables personality profiling through a relatively simple task involving geometric shapes, which enhances its practical utility. For example, it has been used in the development of programs aimed at improving communication skills among university students [11]. Its brevity and ease of administration are expected to further facilitate its use in educational settings such as counseling and mentoring. Although originally developed in South Korea, the tool has been implemented in multiple languages and formats, enabling its potential application across diverse educational and cultural contexts.

#### Need for the present study

Despite the importance of understanding learner characteristics in medical education, little is known about how personality types—as measured by a culturally validated tool such as GEOPIA—are distributed among Korean medical students. Furthermore, no large-scale study has examined whether personality preferences differ according to demographic or academic factors such as gender, age, or stage of training. Such evidence is essential for designing targeted educational interventions, organizing effective learning groups, and strengthening student support strategies in medical education.

### Objectives

This study aimed to (1) identify the distribution of GEOPIA personality types among medical students across multiple institutions; (2) examine whether these personality preferences differ according to gender, age, and academic level; and (3) discuss how GEOPIA profiling may be used to support educational practice in medical school settings.

## Methods

### Ethics statement

The study received ethical approval from the Institutional Review Board (IRB) of Hallym University (IRB No. HIRB-2018–049-1-C). Participation was voluntary, and electronic informed consent was obtained from all participants before the survey. No personal identifiers were collected, and the data were analyzed only in aggregate form.

### Study design

This was a multi-institutional, cross-sectional survey study designed to examine the distribution of GEOPIA personality types among medical students and to explore whether these profiles differ according to demographic characteristics.

### Setting and participants

This questionnaire-based cross-sectional study included students from 5 medical schools in South Korea. Of approximately 40 medical schools nationwide, 5 were selected to reflect regional distribution, including both the Seoul metropolitan area and other regions, as well as variation in school type and institutional size. The selected schools included one public and 4 private institutions, with annual student intakes ranging from approximately 50 to more than 100.

The survey was conducted between 2018 and 2021. Eligible participants were students in years 1 through 6 who were enrolled in required courses during the study period. To minimize self-selection bias, questionnaires were administered during mandatory class sessions. This recruitment strategy allowed us to capture a broad cross-section of the student body with minimal disruption to regular teaching and to obtain the largest practically achievable number of participants at each site.

### Variables

The primary variable of interest was GEOPIA personality type, classified into 4 basic types (Round, Triangle, Box, and Curve) and 12 combined personality profiles based on the first and second preference shapes. Demographic variables included gender, age group, and academic level. Primary and secondary personality types were determined based on the 2 highest GEOPIA dimension scores derived from the item-based test.

### Data sources/measurement

Personality profiles were assessed using the Korean Geometric Psychological Assessment (GEOPIA), a tool developed to measure psychological tendencies using geometric archetypes. The GEOPIA system was developed by Oh [12] at the TNT Human Resources Development Institute in South Korea, and the first version was introduced in 2013. Subsequent validation studies demonstrated acceptable internal consistency (Cronbach’s α=0.894) in Korean populations [12]. In the present study, reliability coefficients for each shape-based scale were 0.895 for Round, 0.917 for Triangle, 0.839 for Box, and 0.852 for Curve, all exceeding the acceptable threshold of 0.7.

GEOPIA has been implemented in multiple formats, including paper-and-pencil and online versions, and has been translated into several languages, including English and Chinese. The assessment consists of 2 components: an item-based test and a drawing-based test. The item-based component evaluates temperament-related personality tendencies, whereas the drawing-based component reflects an individual’s current psychological state. Because the 2 components are not correlated and the aim of this study was to examine temperament-related personality traits, only the item-based test was used.

The item-based test contains 40 items, with 10 items corresponding to each of the 4 geometric shapes. Each item is rated on a 5-point scale and presented as a descriptive word representing a specific personality characteristic. Scores for the 4 shapes are summed, with the highest score indicating the primary preferred shape and the second-highest score indicating the secondary preferred shape. In cases of tied scores, 2 procedures were used to determine the primary and secondary personality types. When available, results from the drawing-based test (GEOGRAM) were used, with the repeatedly drawn “selected shape” serving as the tie-breaker. When drawing-test data were not available, ties were resolved based on item responses by selecting the type with a greater number of highest scores (i.e., 5-point responses). In this study, drawing-test results were available and were used to resolve ties. The drawing-test data are being analyzed separately in another study.

The 4 basic shapes are Round, Triangle, Box, and Curve. Interpretations of the 4 basic types and the 12 combination types are provided in Supplement 1 and Supplement 2. The GEOPIA assessment and demographic questionnaire (gender, age, and academic level) were administered in a paper-and-pencil format. Participation was voluntary, and students completed the questionnaire independently during scheduled class sessions. No identifying information was collected. Data collection was conducted between 2018 and 2021 across the participating medical schools. Because data collection occurred over multiple years, a cross-verification procedure was implemented to prevent duplicate participation.

### Bias

Several potential sources of bias were considered. To reduce self-selection bias, questionnaires were administered during mandatory class sessions whenever possible. Participation remained voluntary, and students completed the questionnaires independently to minimize response influence. In addition, because data were collected over multiple years, a cross-verification procedure was implemented to prevent duplicate participation by the same individuals.

### Study size

The sample size was determined by the maximum achievable number of participants through convenience sampling at 5 medical schools during the study period. A total of 981 students were included in the final analysis, yielding a response rate of 86.6%. Missing data were handled using listwise exclusion. A post hoc power analysis indicated that the sample size (n=981) provided adequate power (>0.80) to detect small-to-medium effects in the chi-square analyses.

### Statistical methods

Descriptive statistics were used to summarize the distribution of GEOPIA personality types. Chi-square (χ2) tests were conducted to examine associations between personality type and gender, age group, and academic level. Statistical significance was set at P<0.05. All analyses were performed using IBM SPSS ver. 29.0.2 (Released 2023; IBM Corp.).

## Results

### Participants

A total of 1,173 students from 5 medical schools were invited to participate. Among them, 1,016 students responded to the survey (response rate, 86.61%), and 981 were included in the final analysis. Of the 35 respondents excluded from the analysis, 25 provided uniform responses across all items, 5 had incomplete responses with missing values on some items, and 5 did not respond to any items. Participant characteristics are presented in Table 1. Male students outnumbered female students by nearly 2 to 1. The distribution of participants across academic years ranged from Year 1 to Year 6.

### Main results

#### Distribution of GEOPIA personality types

Table 2 summarizes the distribution of GEOPIA personality types among medical students. Among the 4 basic personality types, Round was the most prevalent, followed by Box. Triangle and Curve were less frequently identified as primary preferences. Among the combined types, the Round–Box combination was the most common, followed by Box–Round and Round–Triangle.

#### Differences in personality types by gender, age, and academic level

As shown in Table 3, Round was the most prevalent first-preference type in both male and female students, followed by Box. Triangle and Curve showed relatively lower frequencies in both groups. No statistically significant difference in the distribution of GEOPIA basic types was observed by gender.

Across all age groups, the Round and Box types consistently showed higher frequencies than the Triangle and Curve types (Table 4). The largest proportion of respondents was concentrated in the 21–22 and 23–24 age groups across all types. The distribution of GEOPIA basic types did not differ significantly by age.

Across all academic years, Round remained the most common basic type, followed by Box (Table 5). The relative distribution of the 4 basic types showed similar patterns across Years 1 through 6 despite differences in cohort size. No statistically significant differences in GEOPIA basic-type distribution were found according to academic level.

The data used for this study has been provided as Dataset 1.

## Discussion

In this study, Round and Box personality types were the most prevalent among medical students. Among the combined personality profiles, the Round–Box type was the most common, followed by Box–Round and Round–Triangle. The Round–Box profile, often described as the harmonious mediator, reflects interpersonal warmth and empathy combined with responsibility and norm-oriented behavior. In contrast, combinations dominated by Curve and Triangle traits were relatively uncommon, with the Curve–Triangle type appearing least frequently. These findings suggest that medical students tend to exhibit personality profiles characterized by interpersonal harmony and responsibility.

The distribution of GEOPIA basic personality types was also largely consistent across gender, age, and academic level. These findings are consistent with the cohort study by Abbiati and Cerutti [13], which investigated changes in personality traits over the 6 years of medical school among Swiss medical students. Their results showed that personality traits were moderately to highly stable over time, with no significant change observed in most students. Similarly, the findings of the present study suggest that personality traits remain largely stable across academic years rather than differing meaningfully according to level of training. Accordingly, personality traits may be understood less as variables that change over the course of medical school and more as learner characteristics that should be considered when designing educational environments and interventions, including learning support, curriculum design, and student guidance.

Previous studies have reported that personality-related characteristics among medical students are associated with learning behaviors, empathy, communication skills, and professional attitudes [6,14]. The predominance of Round- and Box-oriented profiles observed in this study may therefore reflect interpersonal and responsibility-related tendencies that are often valued in medical training environments. Although developed in South Korea, GEOPIA’s geometric structure allows intuitive cross-cultural interpretation. The availability of English and Chinese versions also suggests that the instrument may have potential applicability in diverse educational contexts internationally.

### Practical advantages of GEOPIA

In Korea, identifying one’s Myers-Briggs Type Indicator (MBTI) type has become a common social practice, reflecting growing interest in rapid and intuitive personality assessment [15]. Compared with the 16 MBTI types, GEOPIA provides a more streamlined framework based on 4 geometric shapes (Round, Triangle, Box, and Curve). One advantage of GEOPIA in educational settings is its brevity; the assessment can be completed in a few minutes and uses descriptive words that are easily understood across age groups.

For medical students and physicians who frequently interact with diverse teams and patients, such simplicity may facilitate quick reflection on interpersonal tendencies [16,17]. Understanding one’s own GEOPIA profile may also promote intuitive awareness of differences in communication and behavioral styles, which can be useful in collaborative learning and clinical environments.

### Individualized counseling and mentoring

Individualized academic guidance should consider not only personality types but also how students perceive their own characteristics and respond to different educational approaches. GEOPIA profiling can help educators identify students’ psychological tendencies and provide a basis for individualized counseling or mentoring.

In the present study, the most prevalent combined type was the Round–Box (○□) profile, characterized by empathy, sociability, and a strong sense of responsibility. Students with this profile may benefit from educational approaches that combine structured academic guidance with relational and emotional support. More broadly, GEOPIA-based counseling may help educators align mentoring strategies with students’ strengths and developmental needs.

### Balanced team-based learning and group dynamics

In student-centered instructional approaches such as team-based learning (TBL), understanding learners’ personality characteristics may facilitate more balanced group composition. The findings of this study indicate that harmony- and responsibility-oriented types (Round and Box) were predominant, whereas leadership-oriented (Triangle) and creativity-oriented (Curve) types were less frequently observed.

Purposefully integrating students with different personality strengths may help optimize group dynamics, promote balanced participation, and enhance collaborative learning outcomes. This perspective is consistent with previous research suggesting that personality traits influence teamwork behaviors and attitudes toward collaboration in medical education [16,17].

### Communication skills and professionalism education

Personality characteristics are closely associated with empathy, interpersonal communication, and professional attitudes [14,18]. Through GEOPIA profiling, students may gain insight into their own psychological tendencies while also learning to recognize diverse communication styles among peers and patients.

Such awareness may contribute to the development of empathy, interpersonal sensitivity, and teamwork competence, which are key elements of professionalism in clinical practice. Reflective exercises, simulated patient encounters, and feedback-based communication training may further support this process.

### Career guidance and specialty exploration

Personality profiling may also support career guidance by helping students reflect on professional pathways that align with their psychological tendencies. Traits such as interpersonal sensitivity, responsibility, leadership orientation, and creativity may influence preferences for particular clinical roles or academic career paths.

Within a broader career counseling framework, GEOPIA may serve as a reflective tool that helps students explore their professional interests and make more informed decisions about their future career development.

### Limitations

Several limitations should be considered when interpreting the findings of this study. First, although South Korea has approximately 40 medical schools, the sample was drawn from only 5 institutions, which limits the generalizability of the results. Second, the number of respondents from higher academic years was relatively small, although no significant cohort differences were observed in the analyses. In addition, while the analysis of the 4 basic personality types was adequately powered, examination of the 12 combined personality types would benefit from a larger sample to improve statistical stability. Although categorizing continuous GEOPIA scores into personality types enhances practical utility for educational counseling and group design, it may also result in a loss of detailed information about the variance and intensity of individual traits.

Furthermore, this study focused primarily on item-based GEOPIA results. Future studies may incorporate data from the drawing-based component to provide a more comprehensive understanding of personality tendencies. Because the present findings are derived from medical students in South Korea, additional research is also needed to examine the cross-cultural validity and applicability of GEOPIA in other educational and cultural contexts.

### Conclusion

This study suggests that GEOPIA profiling offers a feasible and educationally meaningful approach to understanding medical students’ psychological characteristics. By supporting counseling, group-learning design, communication training, and career guidance, GEOPIA may serve as a practical tool for understanding learner characteristics and promoting learner-centered educational practices in medical schools.

## Figures and Tables

**Figure f1-jeehp-23-07:**
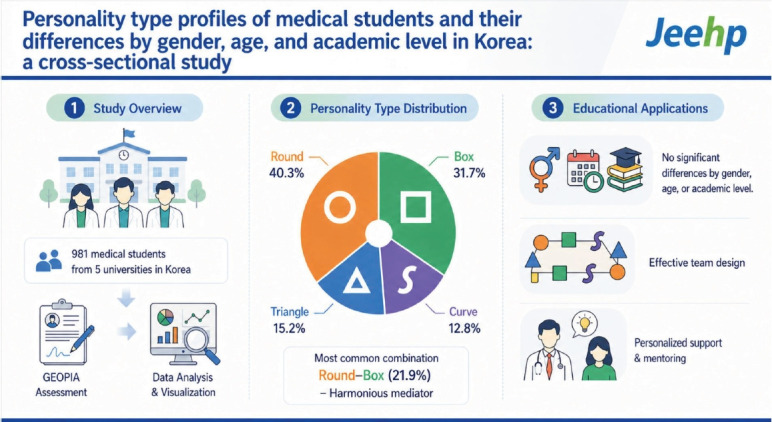


**Table 1.** Participant demographics

Variable	Premedical course		Medical course				Total
	1st	2nd	1st	2nd	3rd	4th	
No. of participants	242 (24.7)	91 (9.3)	435 (44.3)	146 (14.9)	29 (3.0)	38 (3.9)	981 (100.0)
Gender							
Male	172	57	282	82	14	25	632 (66.5)
Female	67	28	142	63	12	7	319 (33.5)
Sub-total	239	85	424	145	26	32	951 (100.0)^a)^
Mean age (yr)	20.7	21.5	22.9	24.0	24.6	26.4	22.5
School							
A	87	56	85	37	-	-	265 (27.0)
B	57	35	37	57	29	-	215 (21.9)
C	98	-	185	-	-	-	283 (28.8)
D	-	-	59	52	-	38	149 (15.2)
E	-	-	69	-	-	-	69 (7.0)

Values are presented as number (%) or number unless otherwise stated.

a) Thirty responses did not include gender.

**Table 2.** Medical students’ personality characteristics of GEOPIA

1st preference type	Frequency (%)	Combination type	Frequency (%)
Round (○)	395 (40.27)	○△	95 (9.74)
		○□	213 (21.85)
		○ S	87 (8.92)
Triangle (△)	149 (15.19)	△○	53 (5.44)
		△□	59 (6.05)
		△ S	34 (3.49)
Box (□)	311 (31.70)	□○	185 (18.97)
		□△	77 (7.90)
		□ S	49 (5.03)
Curve (S)	126 (12.84)	S ○	54 (5.54)
		S △	27 (2.77)
		S □	42 (4.31)
Total	981 (100.00)	Total	975 (100.00)^a)^

GEOPIA, geometric personality assessment tool.

a) Six cases with missing responses for secondary personality type were excluded from the combined-type analysis.

**Table 3.** Differences in GEOPIA based on gender: 4 basic types (%)

Gender	Round (○)	Triangle (△)	Box (□)	Curve (S)	Total
Male	266 (42.1)	87 (13.7)	206 (32.6)	73 (11.6)	632 (100.0)
Female	119 (37.3)	54 (16.9)	95 (29.8)	51 (16.0)	319 (100.0)

χ²=6.360, P=0.095, Cramer’s V=0.082. GEOPIA, geometric personality assessment tool.

**Table 4.** Differences in GEOPIA based on age (%)

Age range (yr)	Round (○)	Triangle (△)	Box (□)	Curve (S)	Total
19–20	58 (16.1)	19 (13.8)	50 (17.2)	13 (10.7)	140 (15.4)
21–22	138 (38.2)	64 (46.4)	112 (38.5)	54 (44.6)	368 (40.4)
23–24	114 (31.6)	33 (23.9)	92 (31.6)	36 (29.8)	275 (30.2)
25–26	35 (9.7)	12 (8.7)	27 (9.3)	14 (11.6)	88 (9.7)
≥27	16 (4.4)	10 (7.2)	10 (3.4)	4 (3.3)	40 (4.4)
Total	361 (100.0)	138 (100.0)	291 (100.0)	121 (100.0)	911 (100.0)

Seventy cases with missing age data were excluded from the analysis. Therefore, age-based analyses were conducted on 911 of the 981 participants. χ²=11.454, P=0.490, Cramer’s V=0.065. GEOPIA, geometric personality assessment tool.

**Table 5.** Distribution of and differences in GEOPIA basic types by academic level

Type	Year 1	Year 2	Year 3	Year 4	Year 5	Year 6
Round (○)	91 (37.6)	39 (42.9)	173 (39.8)	65 (44.5)	12 (41.4)	15 (39.5)
Triangle (△)	47 (19.4)	17 (18.7)	60 (13.8)	18 (12.3)	3 (10.3)	4 (10.5)
Box (□)	78 (32.2)	27 (29.7)	135 (31.0)	43 (29.5)	10 (34.5)	18 (47.4)
Curve (S)	26 (10.7)	8 (8.8)	67 (15.4)	20 (13.7)	4 (13.8)	1 (2.6)
Total	242 (100.0)	91 (100.0)	435 (100.0)	146 (100.0)	29 (100.0)	38 (100.0)

Values are presented as number (%). χ²=18.044, P=0.260, Cramer’s V=0.078. GEOPIA, geometric personality assessment tool.

## Footnotes

**Data availability**
Data files are available from https://doi.org/10.7910/DVN/EIHNEJ Dataset 1. Participants’ demographic data and response data to the GEOPIA item test.

## Supplementary materials

Supplement 1. Interpretation of the 4 basic types and 12 combination types in the GEOPIA item test.

Supplement 2. Characteristics of the 12 combination types in the GEOPIA item test. Adapted with permission from Mira Oh, the copyright holder of the GEOPIA system.

Supplement 3. Audio recording of the abstract.
