# Supplementary material for: Personality type profiles of medical students and their differences by gender, age, and academic level in Korea: a cross-sectional study
Source: J Educ Eval Health Prof. 2026 Apr 28;23:7. doi: 10.3352/jeehp.2026.23.7 (PMC13213262; doi:10.3352/jeehp.2026.23.7)
Supplement: Supplementary file 2 — Supplement 1. Interpretation of the 4 basic types and 12 combination types in the GEOPIA item test. [file jeehp-23-07-suppl1.docx]

Supplement 1. Interpretation of the 4 basic types and 12 combination types in the GEOPIA item test.

| Type | Characteristics of personality types based on the item test | Characteristics |
| --- | --- | --- |
| Round (○) | “A warm-hearted and friendly person”  Empathy, Sociability, Openness | The Round type is an excellent communicator and highly sociable. They are lively and outgoing, possessing a romantic and emotional tendency as a right-brained type. They enjoy storytelling and excel at empathizing within relationships, exhibiting a warm, empathetic, and kind nature. Their qualities make them great counselors, adept at diplomacy and compromise. They also have a keen interest in practical matters and have a natural ability to create a relaxed atmosphere wherever they go, making them natural facilitators. |
| Triangle (△) | “A confident and goal-oriented individual”  Leadership, Initiative, Drive | The Triangle type constantly plans and drives new endeavors. They are proactive and extroverted, with a natural inclination for leadership and entrepreneurship. They are born leaders who strive to achieve high goals and thrive in competitive environments, consistently delivering superior results. When faced with making instant decisions, they do not hesitate and take action. They possess a strong determination to accomplish what they set out to do and demonstrate the courage to restart even after multiple failures. |
| Box (□) | “A meticulous and trustworthy person”  Norms, Responsibility, Effectiveness | The Square types are highly precise and thorough. They are quiet, composed, and calm, instilling a sense of trust. Square types prioritize stability and security, demonstrating a strong practicality and proficiency in handling tasks effectively. However, due to their meticulous and cautious nature, they may be perceived as slow-paced. They possess patience and perseverance, ensuring that once they start a task, they responsibly see it through to the end, even if they may be late in starting. Square types tend to act as stabilizers rather than leaders, and they are often recognized for their consistency and diligence, even though they may not take a prominent role. |
| Curve (S) | “A versatile and free-spirited person”  Flexibility, Creativity, Sensitivity | The Curved type, finds it difficult to tolerate the feeling of being confined within a framework. Introverted curve types are contemplative, meticulous, and delicate. They are versatile and possess excellent skills in handling tools and machinery. They have a refined palate and make excellent gourmet cooks. They are sensitive, pure, and have a great artistic talent. They have distinct likes and dislikes, strong personalities, and unique characteristics. As creative researchers and developers, they are full of ideas and are imaginative and original. They pursue constant change, easily become bored, and require continuous stimulation. They are sensitive to emotions and adapt flexibly according to the situation. |

The table was modified from Oh MR. Development and validation of GEOPIA personality and psychological test using geometry [dissertation]. [Seoul]: Techno-HRD Graduate School, Korea University of Technology Education. 2019. 79p. Korean. Note: Mira Oh is the primary copyright holder of the GEOPIA system; official permission for the reuse and modification of this material has been granted for this publication.
