# Supplementary material for: Personality type profiles of medical students and their differences by gender, age, and academic level in Korea: a cross-sectional study
Source: J Educ Eval Health Prof. 2026 Apr 28;23:7. doi: 10.3352/jeehp.2026.23.7 (PMC13213262; doi:10.3352/jeehp.2026.23.7)
Supplement: Supplementary file 3 — Supplement 2. Characteristics of the 12 combination types in the GEOPIA item test. Adapted with permission from Mira Oh, the copyright holder of the GEOPIA system. [file jeehp-23-07-suppl2.docx]

Supplement 2. Characteristics of the 12 combination types in the GEOPIA item test. Adapted with permission from Mira Oh, the copyright holder of the GEOPIA system.

| Type | Geometry | Symbol | Characteristics |
| --- | --- | --- | --- |
| Round-Triangle  (RT) | ○△ | Sociable Leader | Most extroverted, moody, challenging, intervening, quick-witted, easily excited, and sociable leader |
| Round-Box (RB) | ○□ | Harmonious Mediator | Stable, gentle, warm, kind, submissive, strong sense of belonging, cheerful, lively, passive, unaware of how to refuse |
| Round-Curve (RC) | ○S | Gentle and Warm Leader | Interested in various fields, versatile, emotionally fluctuating, interested in popularity, excellent creativity and critical analysis skills |
| Triangle–Round  (TR) | △○ | Motivated Leader | Strong leadership and drive, diligent and proactive, strong interest and adventurous spirit in everything, clear sense of purpose, always joyful, strong desire for recognition and adept at adapting to circumstances |
| Triangle-Box (TB) | △□ | Systematic Administrator | Long-term planner, administrative temperament, intellectual curiosity, cautious in everything, responsible and diligent, achieves what they desire, strong perseverance |
| Triangle-Curve  (TC) | △S | Adventurous Creator | Goal-oriented, relentless drive in areas of interest, perfectionistic tendencies, good judgment and discernment, strong sense of adventure |
| Box-Round (BR) | □○ | Stable Advisor | Skilled in diplomacy, possessing wisdom and knowledge, outwardly conservative, lack of adventurous spirit, passive, gentle with strong patience, thoughtful and kind |
| Box-Triangle (BT) | □△ | Cautious and Objective Leader | Objective and pragmatic, level-headed, achievement-oriented, committed to completing tasks, strong conviction and stubbornness, somewhat passive and calm, sense of responsibility, cautious. |
| Box-Curve  (BC) | □S | Perfectionistic Knowledge Seeker | Dignified and elegant, cautious and meticulous, attention to detail, perfectionistic tendencies, strong inner beliefs, strong desire for belonging, tendency to procrastinate tasks |
| Curve-Round (CR) | S○ | Emotional Artist | Naturally artistic sense, keen interest in the arts, emotional, good interpersonal skills, insight and judgment, kind-hearted, low self-esteem, easily hurt |
| Curve-Triangle  (CT) | S△ | Creative Idea Bank | Creative planning ability, drive and determination, leadership skills in the fields of arts, culture, and sports, cautious and focused, thorough preparation |
| Curve-Box  (CB) | S□ | Calm and Cautious Artist | Most introverted, quiet and cautious, thoughtful, inclined towards perfecting things, conscientious, prone to inner conflicts, easily stressed type, tendency to underestimate one's own abilities |

R, round; T, triangle; B, box; C, curve

The table was modified from Oh M. 12 gaji gijilgwa dohyeong simni. In: Oh M. Dohyeongsimniro ttongthaneun gwangyesimnihak: Psychology-Geometrics System. Seoul: Bukself. 2011, pp. 103-113. Korean. Note: Mira Oh is the primary copyright holder of the GEOPIA system; official permission for the reuse and modification of this material has been granted for this publication.
